# Improvement in Ionization Efficiency Using Metal Oxide Nanoparticles in Laser Desorption/Ionization Mass Spectrometry of a Cancer Drug

**DOI:** 10.5702/massspectrometry.A0099

**Published:** 2021-12-23

**Authors:** Hiroki Kannen, Yuto Miyoshi, Hisanao Hazama, Kunio Awazu

**Affiliations:** 1 Graduate School of Engineering, Osaka University, 2–1 Yamadaoka, Suita, Osaka 565–0871, Japan; 2 Global Center for Medical Engineering and Informatics, Osaka University, 2–2 Yamadaoka, Suita, Osaka 565–0871, Japan

**Keywords:** nanoparticle, laser desorption/ionization, mass spectrometry imaging, cancer drug, ionization efficiency

## Abstract

Mass spectrometry imaging (MSI) without labeling has the potential for faster screening in drug development. Matrix-assisted laser desorption/ionization (MALDI) is typically used, but it has a large matrix size and uneven drug distribution. Surface-assisted laser desorption/ionization (SALDI) using nanoparticles (NPs) may overcome these issues. Here, the influence of NPs, solvent ratio, and order of dropping of NPs on SALDI-MSI of protoporphyrin IX (PpIX), a cancer drug, are reported. A solution of PpIX in a 50% aqueous solution of 50% acetonitrile at a concentration of 10 μM was used. The NPs include ZnO, Fe_3_O_4_, and four types of TiO_2_. The NPs were fabricated by dissolving them on an aqueous 90% acetonitrile solution. Mass spectra were obtained with a time-of-flight mass spectrometer using a Nd:YAG laser at a 355-nm wavelength. The signal intensity using TiO_2_ at a 0.5 mg/mL concentration in 50% acetonitrile was increased by 1.6-fold compared to that without TiO_2_. Changing the solvent to 90% acetonitrile gave a uniform TiO_2_ distribution and a 9-fold increase in the signal intensity for PpIX. Among the four types of TiO_2_ with different particle sizes and crystal structures, TiO_2_ with a smaller particle size and a rutile crystal structure produced the highest signal intensity. Forming a layer on top of the PpIX also resulted in an increased signal intensity. Hence, SALDI using TiO_2_ provides effective ionization of the drug. In the future, we plan to investigate a spray method for the ionization of PpIX using TiO_2_ for the MSI of various drugs.

## INTRODUCTION

More than 10,000 candidates are generally required to develop one new drug. As such, the research and development period for a new drug is currently 10–15 years and the average cost is more than 800 million dollars.^1)^ Thus, enhancing the productivity of new drug development would be highly desirable. Screening is used to identify a target substance from a huge number of candidates in the early stages of drug development. High-throughput screening, which can comprehensively evaluate the efficacy, safety, and pharmacokinetics of a drug from the initial stage of development, is important.^2,3)^

A faster screening method would clearly facilitate drug development. Currently, autoradiography is the most common method for drug screening,^4)^ but it has drawbacks such as increased costs due to labeling, long measurement times, and the need for the simultaneous measurements of metabolites. On the other hand, mass spectrometry imaging (MSI)^5,6)^ does not require labeling, which is advantageous in terms of cost and measurement time. In matrix-assisted laser desorption/ionization (MALDI), which is mainly used in MSI, aromatic compounds with an absorption peak in the ultraviolet region are widely used as ionization-assistant reagents.^7–9)^ It has been noted that, when the matrix is sprayed onto the sample, the crystal size of the matrix becomes large, and the drug distribution becomes uneven.

In recent years, surface-assisted laser desorption/ionization (SALDI) has been attracting interest as a potential solution to this problem.^10–12)^ In SALDI, metal oxide nanoparticles (NPs), which have absorption peaks in the ultraviolet region, are used as reagent for assisting the ionization process. In this method, the sample molecules adhere to the surface of the NPs and electrons or cations are then generated from the NPs electronically by excitation with a laser instead of the process in which the sample molecules and the matrix form mixed crystals. The crystals act like a matrix and may reduce the disorder of drug distribution. In addition, since matrix-derived ions are not detected in the small molecule region, the analysis of small molecule samples is facilitated.^13)^ To date, relatively high molecular weight lipids have been analyzed *via* SALDI, but there are few studies in which the types, particle sizes, concentrations, and surface conditions of NPs have been examined for MSI of drugs.^14)^ In this study, we report on the application of SALDI to drugs in an attempt to improve ionization efficiency.

## EXPERIMENTAL

### Time-of-flight mass spectrometer

All experiments were performed using a time-of-flight (TOF) mass spectrometer (Voyager DE-PRO; Applied Biosystems, Foster, CA, USA) equipped with a 355-nm third-harmonic Nd:YAG laser (GAIA II 30-T; Rayture Systems, Tokyo, Japan). The instrument parameters in the reflectron-mode of Voyager DE-PRO were set as follows: +20 kV acceleration voltage, +13.6 kV voltage for the extraction grid, 0 V for the guide wire, and 100 ns extraction delay time.

### Scanning Electron Microscope

A scanning electron microscope (SEM, JCM-5700; JEOL, Tokyo, Japan) was used to observe the distribution of NPs in the sample spot.

### Sample preparation

Protoporphyrin IX (PpIX, P8293; SIGMA-Aldrich, Tokyo, Japan), which has basic skeleton of hemoglobin, is also used as a cancer treatment drug and in photodynamic therapy (PDT). Although it is metabolized in normal tissue, it accumulates in cancer cells due to a metabolic abnormality.^15)^ In this study, PpIX is used as the cancer drug.

We investigated three types of metal oxide NPs: TiO_2_, ZnO (APR5350; Wako, Osaka, Japan), Fe_3_O_4_ (MKCM9976; SIGMA-Aldrich). These NPs effectively ionize small molecules in samples.^16–18)^ In addition, three other types of TiO_2_, with different sizes and crystal structures, were evaluated. The particle sizes and crystal structures of the four types of TiO_2_ were 280 nm and rutile (CR-58; Ishihara Sangyo Kaisha, Osaka, Japan), 21 nm and anatase/rutile (718467-100G; SIGMA-Aldrich), 5–15 nm and rutile (US7050; US Research Nanomaterials, Houston, TX, USA), and 5 nm and anatase (7930DL; Skyspring Nanomaterials, Houston, TX, USA). The particle sizes, crystal structures and other information concerning the NPs can be found on the websites of purchasers mentioned above.

We examined two application methods of NPs with different matrix application orders. While dropping the matrix on the drug is a common approach in imaging, dropping the drug on the matrix may also be a feasible approach. Herein the NPs were applied under the sample so that the ionization efficiency was relatively high. After investigating sample preparation methods, the NPs were dropped onto the sample for MSI.

In this experiment, a metal plate (4347686; Applied Biosystems, Foster City, CA, USA) was used as the sample plate.

## RESULTS AND DISCUSSION

### Detection of PpIX using NPs

To investigate the efficiency of NPs for the ionization of PpIX, we measured PpIX in a mixture, which was prepared by mixing PpIX in water with 50% acetonitrile at a concentration of 10 μM. The NPs were dissolved in water with 50% acetonitrile at concentrations of 0, 0.1, 0.5, 1, and 5 mg/mL. In this experiment, the TiO_2_ particles had a particle size of 21 nm and each NP was sonicated for 20 min. NPs with a volume of 1 μL were dropped onto the metal plate which was then dried in a vacuum. A mixture of pure PpIX in 1 μL of solution was dropped on the dried spot containing the NPs and mass spectra were obtained with a TOF mass spectrometer. A laser was randomly irradiated at 10 points for one spot and 100 pulses per point and the respective spectra were then averaged to obtain one mass spectrum.

**Figure 1:**
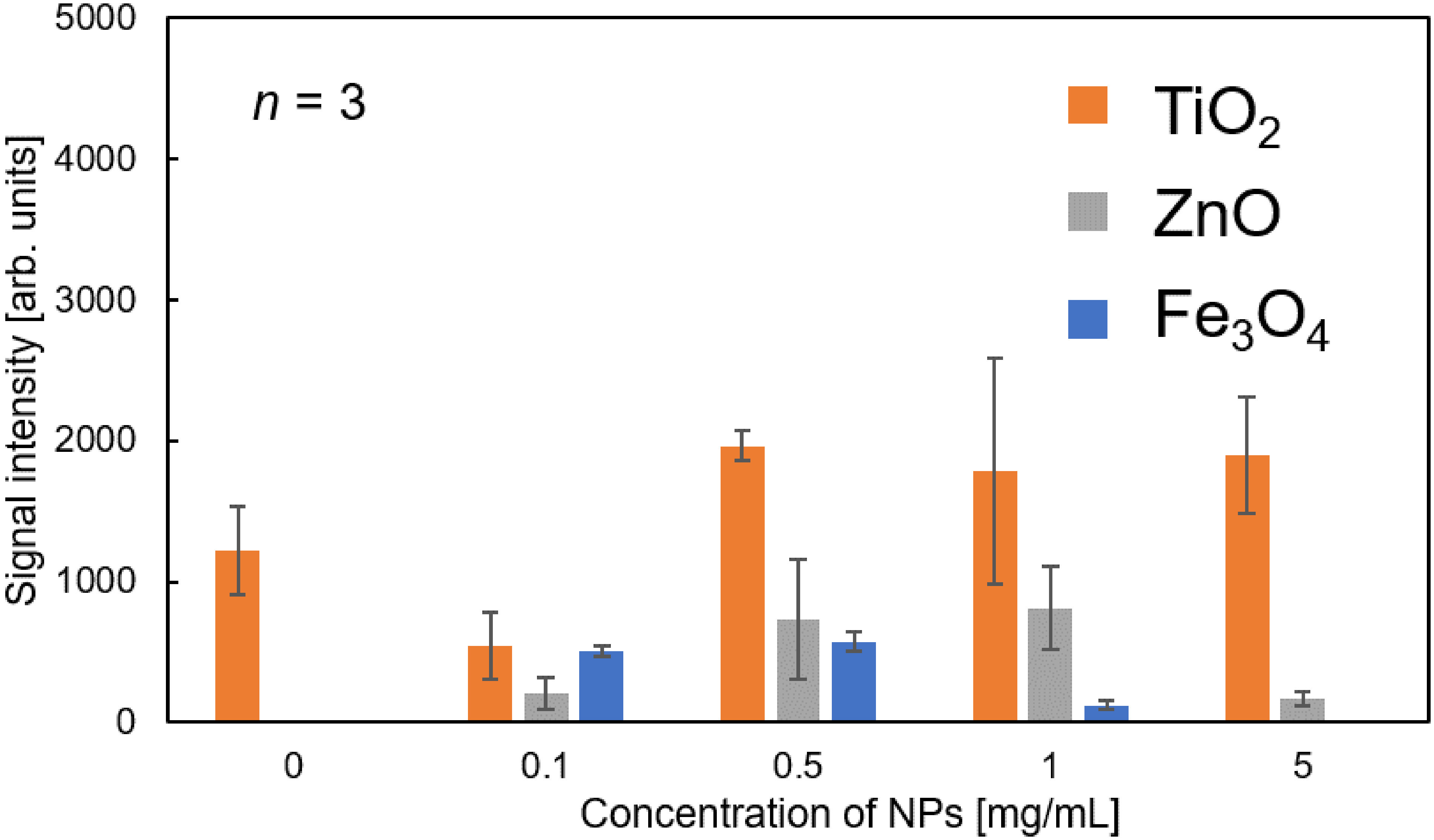
Fig. 1. Typical mass spectra obtained from a mixture of pure PpIX in a volume of 1 μL using (a) no NPs, (b) TiO_2_, (c) ZnO, and (d) Fe_3_O_4_. PpIX was dropped on each NP. Particle size of TiO_2_ is 21 nm.

**Figure 2:**
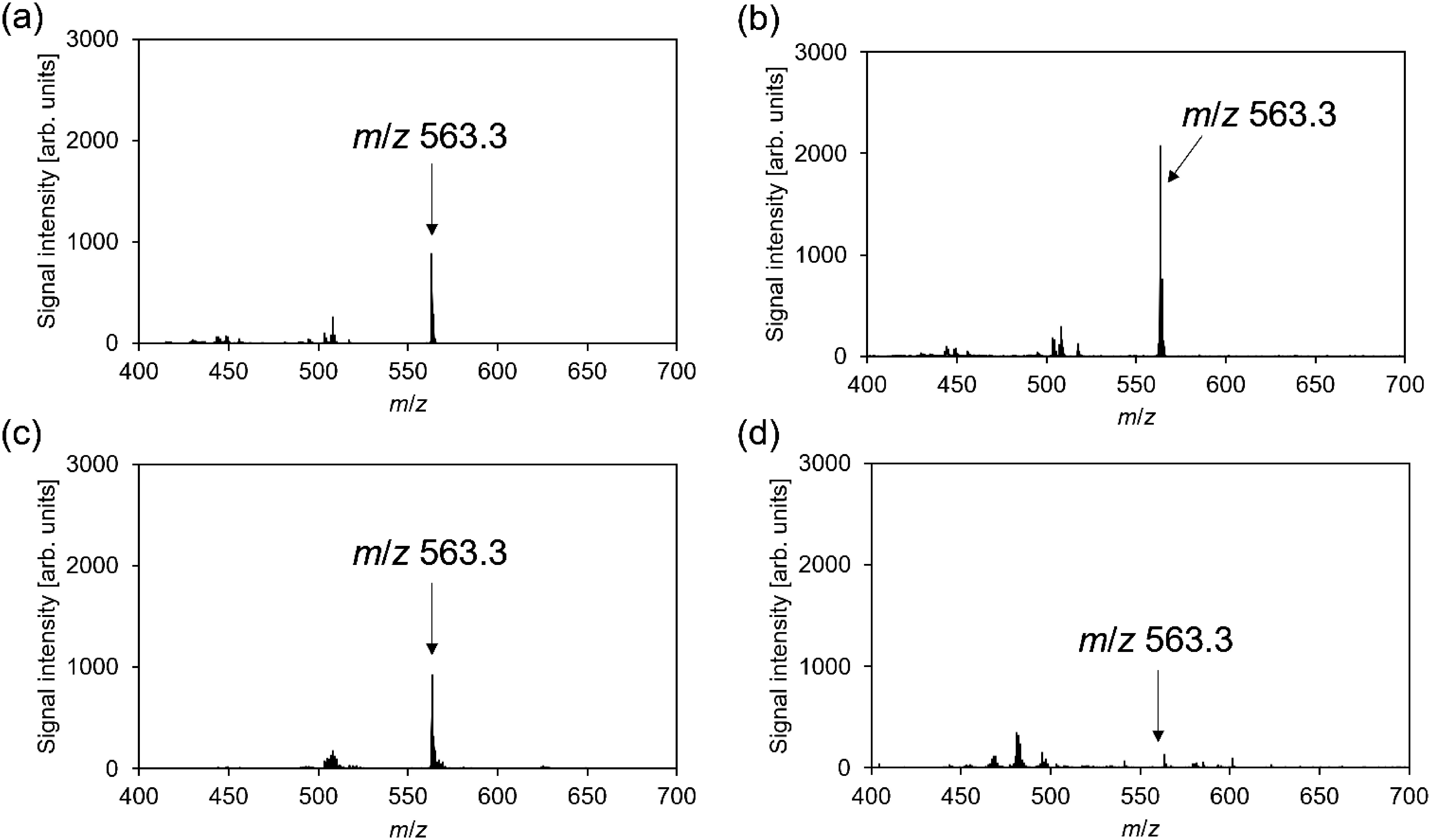
Fig. 2. Average signal intensities of the ions at *m*/*z* 563.3 obtained from a mixture of pure PpIX using NPs at concentrations of 0, 0.1, 0.5, 1, and 5 mg/mL. The particle size of TiO_2_ was 21 nm.

Figure 1 shows typical mass spectra obtained from a mixture of pure PpIX with a volume of 1 μL using (a) no NPs, (b) TiO_2_, (c) ZnO, and (d) Fe_3_O_4_ at a concentration of 0.5 mg/mL. Compared to the case without NPs, the signal intensity of the ion at *m*/*z* 563.3 increased when TiO_2_ was used. Figure 2 shows the average signal intensities of the ions at *m*/*z* 563.3 obtained from a mixture of pure PpIX and different NP concentrations (0, 0.1, 0.5, 1, and 5 mg/mL). a concentration of 0 mg/mL of NPs denote the results obtained when only PpIX was examined in all figures. Using TiO_2_ as reagent for assisting ionization enhanced the sensitivity of detection of the ions at *m*/*z* 563.3 by 1.6-fold compared to PpIX only. Although it has been reported that ZnO and Fe_3_O_4_ are effective in the ionization of small molecule samples and peptides,^16,17)^ the signal intensity of PpIX tended to decrease when these NPs were used. We conclude that the ionization efficiency of the sample differs depending on the chemical properties of the reagents being used in SALDI.

### Investigation of the solvent ratio for TiO_2_

TiO_2_ was found to improve the ionization efficiency of PpIX among NPs. In SALDI, the ionization efficiency depends on the state of aggregation in the dried sample. Thus, we investigated the influence of the matrix solvent on ionization efficiency and aggregation state.

A mixture of pure PpIX in water with 50% acetonitrile at a concentration of 10 μM was prepared. TiO_2_ particles with a size of 21 nm were made by dissolving it in water with 10%, 50%, or 90% acetonitrile at a concentration of 0, 1, or 5 mg/mL. Increasing the proportion of acetonitrile above 90% caused the spots of the dropped TiO_2_ to expand, making sample preparation difficult. Each TiO_2_ sample was sonicated for 20 min. TiO_2_ in a volume of 1 μL was dropped onto a metal plate and dried in a vacuum and PpIX was then dropped on the dried TiO_2_ spot.

**Figure 3:**
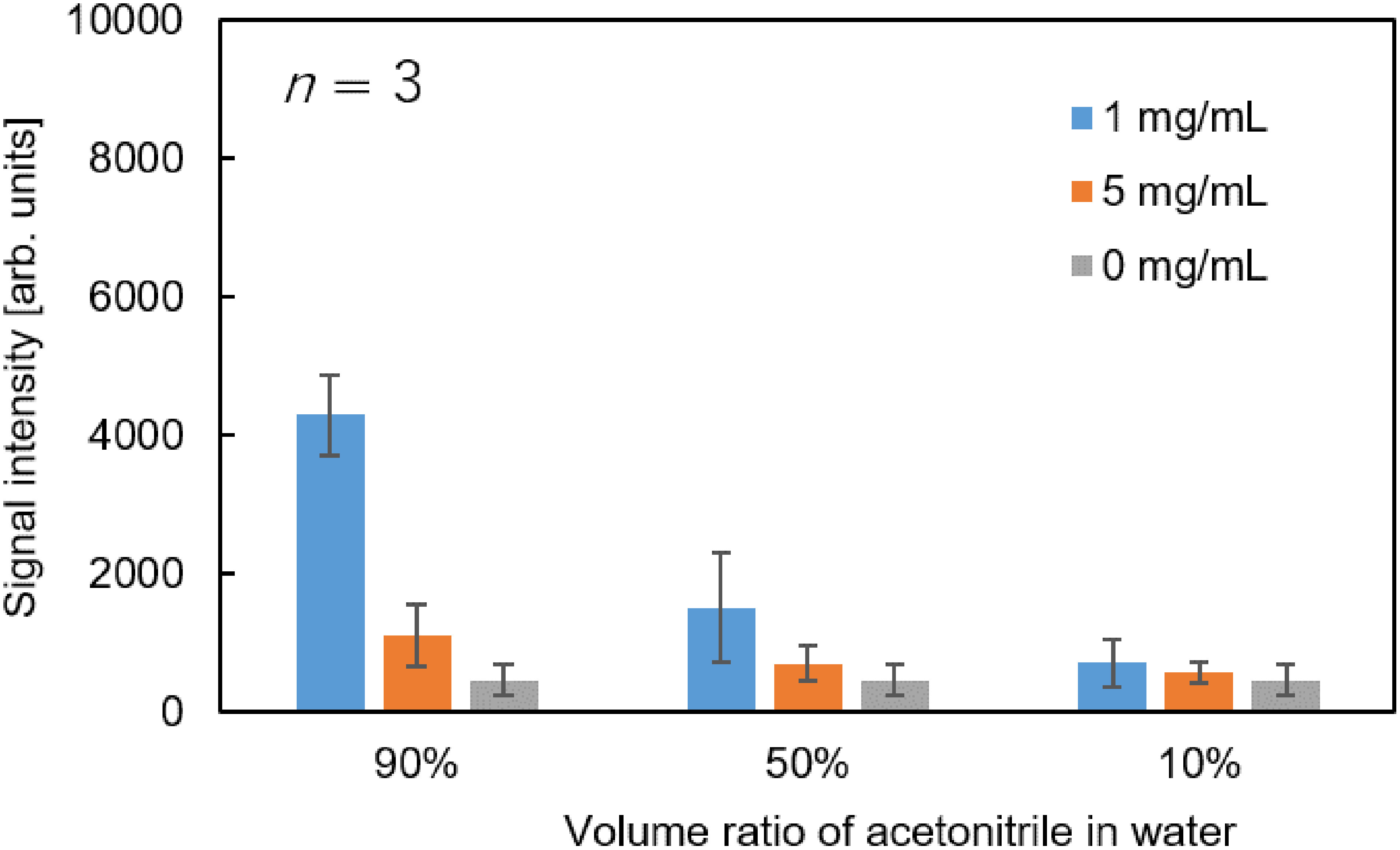
Fig. 3. Average signal intensities of ions at *m*/*z* 563.3 with TiO_2_ at concentrations of 0, 1, and 5 mg/mL using 10%, 50%, or 90% acetonitrile as the solvent. PpIX was dropped on the TiO_2_. The particle size of TiO_2_ was 21 nm.

Figure 3 shows the average signal intensities for PpIX with TiO_2_ at concentrations of 0, 1, and 5 mg/mL in water with 10%, 50%, or 90% acetonitrile. A 90% aqueous acetonitrile solution resulted in the highest signal intensity. The signal intensity of PpIX increased with increasing ratio of acetonitrile. The signal intensity was about 12.7-fold higher than that without TiO_2_.

**Figure 4:**
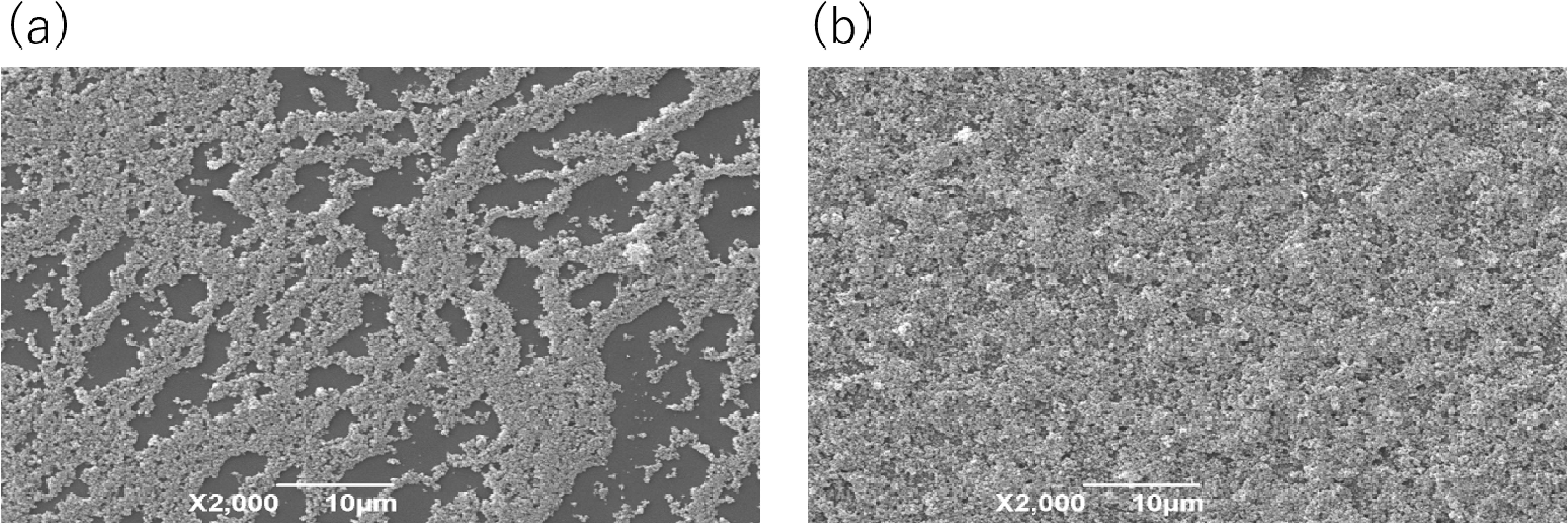
Fig. 4. SEM images obtained from dropping spots of TiO_2_ dissolved in water with (a) 10% and (b) 90% acetonitrile at a concentration of 1 mg/mL.

Figure 4 shows SEM images obtained from dropping spots of TiO_2_ at a concentration of 1 mg/mL dissolved in water with 10% or 90% acetonitrile. The NPs were more uniformly distributed in the spots in the case of a 90% acetonitrile aqueous solution compared to a 10% acetonitrile aqueous solution. Since acetonitrile is more volatile than water, a homogeneous layer of TiO_2_ would be formed. The data shown in Figs. 3 and 4 indicate that when the proportion of acetonitrile is increased, the distribution of NPs becomes uniform and the signal intensity of PpIX increases because it is less affected by aggregation.

### Comparison of ionization efficiency using four types of TiO_2_

In this experiment, we investigated the sensitivity of detection of PpIX using four types of TiO_2_. A mixture was prepared of pure PpIX in water with 50% acetonitrile at a concentration of 10 μM. The four types were prepared by dissolving TiO_2_ in water with 90% acetonitrile at concentrations of 0, 0.1, 0.5, 1, 5, and 10 mg/mL. Each TiO_2_ sample was sonicated for 20 min and a volume of 1 μL was then dropped onto a metal plate and dried in a vacuum, after which, the PpIX was dropped on the dried spot of TiO_2_.

**Figure 5:**
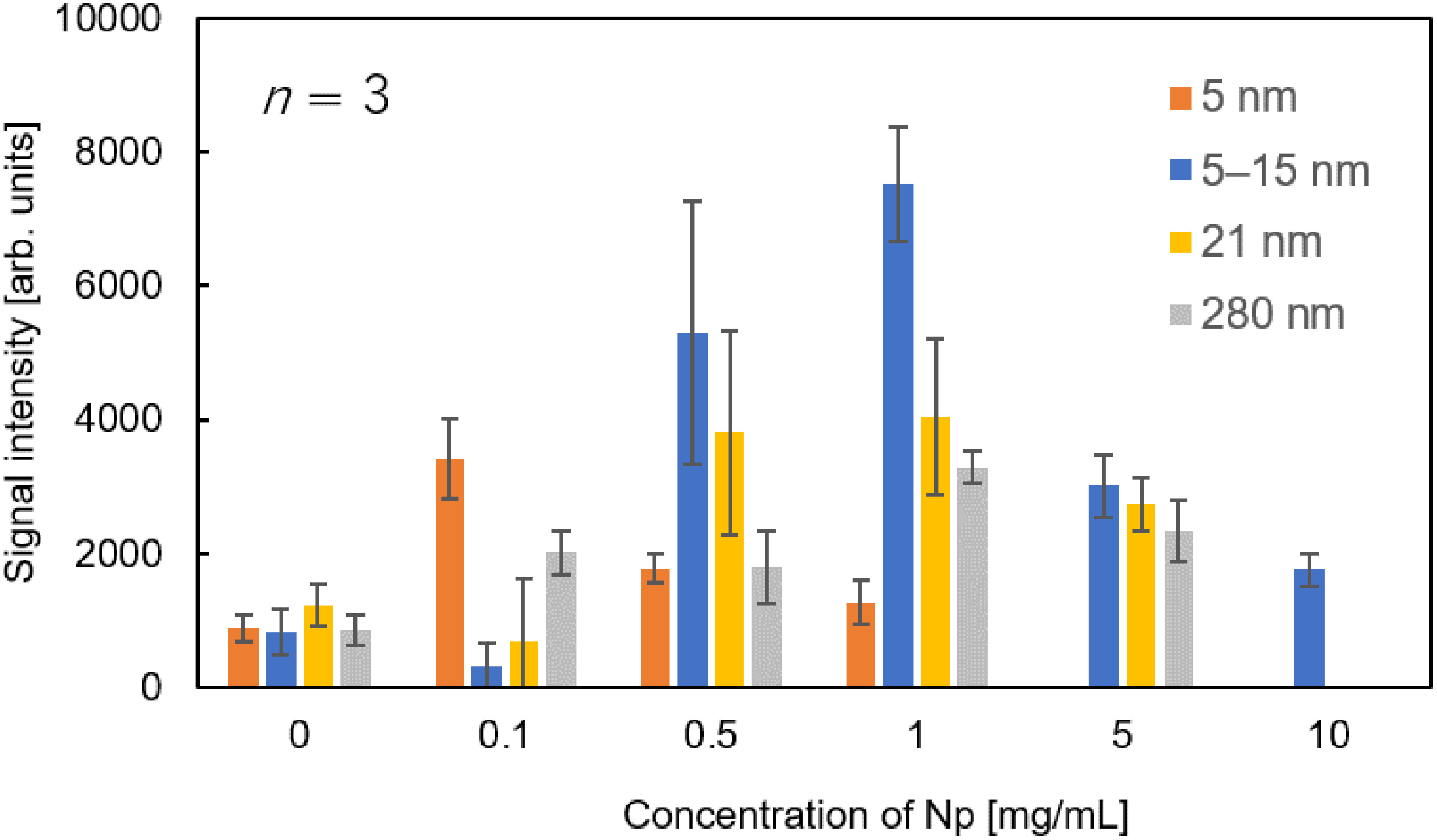
Fig. 5. Average signal intensities of ions at *m*/*z* 563.3 using four types of TiO_2_ at concentrations of 0–10 mg/mL. The PpIX was dropped on the TiO_2_.

Figure 5 shows the average signal intensities of PpIX when the four types of TiO_2_ were used. The highest signal intensity was obtained when 5–15 nm TiO_2_ was used. The signal intensity was about 9.0-fold higher than that obtained from PpIX only. A peak corresponding to protonated PpIX was not obtained when 280-nm TiO_2_ was used at concentrations of 5 and 10 mg/mL. This suggests that the signal intensity decreases with increasing particle size when high concentrations of TiO_2_ are used. The signal intensity was higher when 5–15-nm TiO_2_ was used compared to the use of 5-nm TiO_2_. This difference can be attributed to the crystal structure. TiO_2_ with a particle size of 5–15 nm has a rutile crystal structure. Generally, in SALDI, a rapid temperature increase in the pulse laser irradiation greatly affects the efficiency of sample ionization. The UV absorption efficiency of rutile-type TiO_2_ is higher than that of the anatase-type.^19)^ Therefore, the signal intensity obtained from PpIX using 5–15 nm TiO_2_, which has a rutile-type crystal structure and a small particle size, was the highest.

### Comparison of ionization efficiency by order of dropping of NPs

In the above experiments, PpIX was dropped onto NPs. In MSI, it is necessary to form a layer of NPs on the measurement sample. The effect of the order of dropping on the detection sensitivity was investigated.

A mixture of pure PpIX was prepared in water with 50% acetonitrile at a concentration of 10 μM. TiO_2_ NPs with a particle size of 21 nm were prepared by dissolving TiO_2_ in water with 90% acetonitrile at a concentration of 0, 0.1, 0.5, 1, or 5 mg/mL. Each TiO_2_ sample was sonicated for 20 min. PpIX or TiO_2_ with a volume of 1 μL was dropped onto a metal plate, dried in a vacuum and TiO_2_ or PpIX was then dropped on the dried spot.

**Figure 6:**
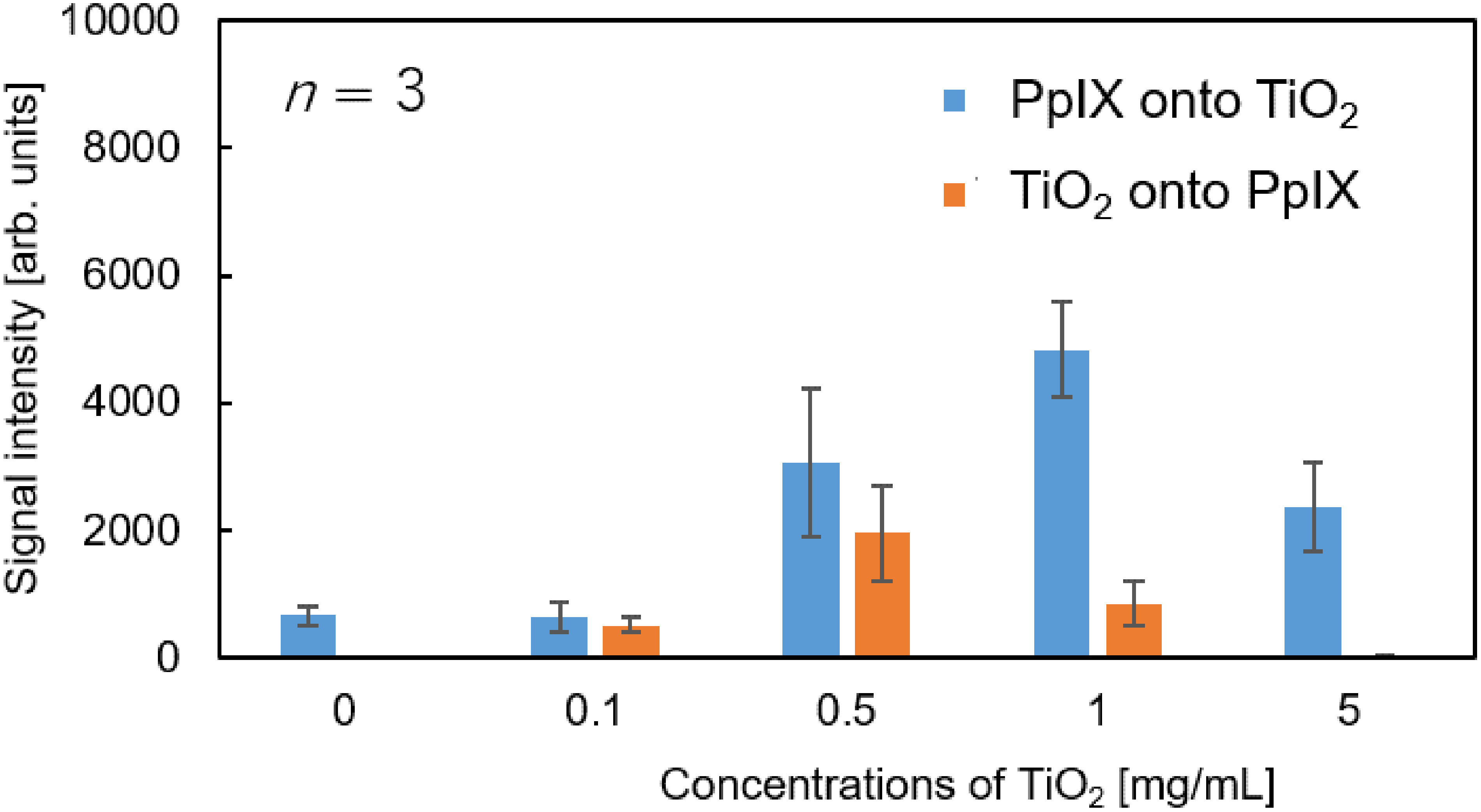
Fig. 6. Average signal intensities of ions at *m*/*z* 563.3 with TiO_2_ at a concentration of 1 mg/mL using 90% acetonitrile as the solvent in two dropping orders of TiO_2_. The particle size of TiO_2_ is 21 nm.

Figure 6 shows the average signal intensities for PpIX with TiO_2_ at concentrations of 0, 0.1, 0.5, 1, and 5 mg/mL using 90% acetonitrile as the solvent for the two dropping orders. When a layer of NPs was formed on top of the PpIX, the signal intensity was reduced. This tendency was more pronounced as the concentration of TiO_2_ was increased. In SALDI, NPs absorb energy from the laser irradiation and then transfer it efficiently to the analyte. When TiO_2_ is dropped onto the sample, the energy is absorbed by the layer of TiO_2_ on the surface of the sample, and it is generally assumed that the efficiency of energy transfer to PpIX decreases. Further, regardless of the dropping order, when the concentration of TiO_2_ becomes too high, the signal intensity is lowered because the NPs undergo aggregation. The signal intensity was the highest when TiO_2_ was dropped on PpIX at a concentration of 0.5 mg/mL and was about 3-fold higher than the value without PpIX. The spot diameter of TiO_2_ was about 1 mm. The estimated suitable density of TiO_2_ for ionization of PpIX was about 0.64 μg/mm^2^.

For MSI, an ionization-assisting reagent was applied on the sample using various methods.^20–22)^ In future work, we plan to investigate the spray method for the ionization of PpIX using TiO_2_.

## CONCLUSION

For SALDI-MSI of a cancer drug using NPs, we investigated different factors: the type of NPs used, solvent ratio, and dropping order for the ionization of pure PpIX. In initial experiments, we examined the ionization efficiencies of PpIX using TiO_2_, ZnO, and Fe_3_O_4_, which are appropriate for the ionization of samples containing small molecule. The signal intensity using TiO_2_ with 50% acetonitrile at a concentration of 0.5 mg/mL was 1.6-fold higher than that without TiO_2_. In addition, changing the solvent for the TiO_2_ to 90% acetonitrile resulted in the uniform distribution of TiO_2,_ and the signal intensity of PpIX was increased 9-fold. The signal intensity of PpIX tended to decrease when ZnO and Fe_3_O_4_ were used in this experiment. These NPs were found to be unsuitable for the ionization of PpIX. We next investigated four types of TiO_2_ samples with different particle sizes and crystal structures. The highest signal intensity for PpIX was obtained using TiO_2_ with a 5–15 nm particle size and a rutile crystal structure. The signal intensities of PpIX were compared for different orders of dropping of TiO_2_ for MSI. When a layer of NPs was formed on top of the PpIX, the ionization efficiency decreased. However, the signal intensity was about 3-fold higher than that for PpIX only. These results demonstrate that the use of TiO_2_ in SALDI can be used to effectively measure PpIX. In the future, we plan to investigate the spray method for the ionization of PpIX using TiO_2_ for the MSI of various drugs.

## Notes

Mass Spectrom (Tokyo) 2021; 10(1): A0099

## References

[1] K. Shimada. Pharmacokinetic research in the early stages of drug discovery: Significance and practice. *Folia Pharmacol. Jpn.* 133: 210–213, 2009. 10.1254/fpj.133.210 19367023

[2] I. Horii. Safety, pharmacokinetic, and physicochemical studies in the early stage of drug discovery. *Folia Pharmacol. Jpn.* 127: 217–221, 2006. 10.1254/fpj.127.217 16651807

[3] Y. Yamada. High-throughput analysis technique in the drug discovery stage. *Folia Pharmacol. Jpn.* 135: 109–112, 2010. 10.1254/fpj.135.109 20228575

[4] J. M. Chirgwin, A. E. Przybyla, R. J. MacDonald, W. J. Rutter. Isolation of biologically active ribonucleic acid from sources enriched in ribonuclease. *Biochemistry* 18: 5294–5299, 1979. 518835 10.1021/bi00591a005

[5] S. Shimma, M. Setou. Applications of a mass microscope for bionanotechnology. *Hyomen Kagaku* 27: 79–85, 2006.

[6] S. Shimma, M. Setou. Review of imaging mass spectrometry. *J. Mass Spectrom. Soc. Jpn.* 53: 230–238, 2005.

[7] Y. Dai, R. M. Whittal, L. Li. Two-layer sample preparation: A method for MALDI-MS analysis of complex peptide and protein mixtures. *Anal. Chem.* 71: 1087–1091, 1999. 10079766 10.1021/ac980684h

[8] K. Gevaert, J. Vandekerckhove. Protein identification methods in proteomics. *Electrophoresis* 21: 1145–1154, 2000. 10786887 10.1002/(SICI)1522-2683(20000401)21:6<1145::AID-ELPS1145>3.0.CO;2-Z

[9] Z. Y. Park, D. H. Russell. Identification of individual proteins in complex protein mixtures by high-resolution, high-mass-accuracy MALDI TOF-mass spectrometry analysis of in-solution thermal denaturation/enzymatic digestion. *Anal. Chem.* 73: 2558–2564, 2001. 11403300 10.1021/ac001488p

[10] R. Arakawa, H. Kawasaki. Functionalized nanoparticles and nanostructured surfaces for surface-assisted laser desorption/ionization mass spectrometry. *Anal. Sci.* 26: 1229–1240, 2010. 21157090 10.2116/analsci.26.1229

[11] T. Guinan, P. Kirkbride, P. E. Pigou, M. Ronci, H. Kobus, N. H. Voelcker. Surface-assisted laser desorption ionization mass spectrometry techniques for application in forensics. *Mass Spectrom. Rev.* 34: 627–640, 2015. 24916100 10.1002/mas.21431

[12] H. W. Tang, K. M. Ng, W. Lu, C. M. Che. Ion desorption efficiency and internal energy transfer in carbon-based surface-assisted laser desorption/ionization mass spectrometry: Desorption mechanism(s) and the design of SALDI substrates. *Che. Anal. Chem.* 81: 4720–4729, 2009. 19449861 10.1021/ac8026367

[13] D. S. Peterson. Matrix-free methods for laser desorption/ionization mass spectrometry. *Mass Spectrom. Rev.* 26: 19–34, 2007. 16967450 10.1002/mas.20104

[14] T. Yonezawa, T. Asano, M. Matsubara. Surface-assisted laser desorption ionization mass spectrometry (SALDI-MS) of low-molecular-weight medicines and toxic materials using commercial TiO_2_ nanoparticles. *Chem. Soc. Jpn.* 89: 346–353, 2016.

[15] M. Yamauchi, N. Honda, H. Hazama, S. Tachikawa, H. Nakamura, Y. Kaneda, K. Awazu. A novel photodynamic therapy for drug-resistant prostate cancer cells using porphyrus envelope as a novel photosensitizer. *Photodiagnosis Photodyn. Ther.* 11: 48–54, 2014. 24629697 10.1016/j.pdpdt.2013.10.001

[16] C. K. Chiang, N. C. Chiang, Z. H. Lin, G. Y. Lan, Y. W. Lin, H. T. Chang. Nanomaterial-based surface-assisted laser desorption/ionization mass spectrometry of peptides and proteins. *J. Am. Soc. Mass Spectrom.* 21: 1204–1207, 2010. 20430643 10.1016/j.jasms.2010.02.028

[17] G. Gedda, H. N. Abdelhamid, M. S. Khan, H. F. Wu. ZnO nanoparticle-modified polymethyl methacrylate-assisted dispersive liquid–liquid microextraction coupled with MALDI-MS for rapid pathogenic bacteria analysis. *RSC Advances* 4: 45973–45983, 2014.

[18] K. Shrivas, T. Hayasaka, Y. Sugiura, M. Setou. Method for simultaneous imaging of endogenous low molecular weight metabolites in mouse brain using TiO_2_ nanoparticles in nanoparticle-assisted laser desorption/ionization-imaging mass spectrometry. *Anal. Chem.* 83: 7283–7289, 2011. 21894964 10.1021/ac201602s

[19] H. Kawasaki, K. Okumura, R. Arakawa. Influence of crystalline forms of titania on desorption/ionization efficiency in titania-based surface-assisted laser desorption/ionization mass spectrometry. *J. Mass Spectrom. Soc. Jpn.* 58: 221–228, 2010.

[20] Y. Sugiura, S. Shimma, M. Setou. Two-step matrix application technique to improve ionization efficiency for matrix-assisted laser desorption/ionization in imaging mass spectrometry. *Anal. Chem.* 78: 8227–8235, 2006. 17165811 10.1021/ac060974v

[21] J. A. Hankin, R. M. Barkley, R. C. Murphy. Sublimation as a method of matrix application for mass spectrometric imaging. *J. Am. Soc. Mass Spectrom.* 18: 1646–1652, 2007. 17659880 10.1016/j.jasms.2007.06.010 PMC2042488

[22] S. Shimma. Characterizations of two-step matrix application procedures for imaging mass spectrometry. *Mass Spectrom. Lett.* 6: 21–25, 2015.

